# Quality of Life: Updated Psychometric Properties and New Norm Values in a Representative German Sample Focusing Socioeconomics and Mental Health

**DOI:** 10.3389/ijph.2022.1605188

**Published:** 2022-10-05

**Authors:** Nora Hettich, Manfred E. Beutel, Lina Krakau, Elmar Braehler

**Affiliations:** ^1^ University Medical Centre, Johannes Gutenberg University Mainz, Mainz, Germany; ^2^ Integrated Research and Treatment Center, University Hospital Leipzig, Leipzig, Germany

**Keywords:** general population, quality of life, psychometric properties, EUROHIS-QOL, norms, PHQ-4

## Abstract

**Objectives:** Quality of life (QOL) is increasingly used as indicator in health research. The aim of this paper was an updated psychometric validation and a new standardization of the German version of the EUROHIS-QOL using a sample of the German general population assessed in 2021. The study focused on socio-economic characteristics and on anxiety and depressiveness as major indicators of mental health.

**Methods:** With 8 items, the EUROHIS-QOL is an economical instrument for self-assessment.

**Results:** Statistical tests revealed good psychometric properties. Gender- and age-group-specific norm values were calculated. The EUROHIS-QOL showed good discriminant validity for anxiety and depression symptoms. Participants without clinically relevant scores for depressiveness and anxiety reported significantly higher QOL. Multiple regression analysis showed that unemployment, younger age, not living with a partner, and an immigrant background were important predictors of lower QOL, whereas higher income, living in one’s own home, and a high level of education predicted higher QOL.

**Conclusion:** The EUROHIS-QOL was confirmed as an economical and reliable instrument for assessing QOL in the German general population.

## Introduction

Beyond health-related quality of life the concept of general quality of life (QOL) is increasingly used as an important indicator in health research. The World Health Organization (WHO) and the WHOQOL group defined QOL as an “individual’s perception of their position in life in the context of the culture and value systems in which they live and in relation to their goals, expectations, standards and concerns [1].” This definition broadens the perspective from health-related QOL to a more comprehensive and complex concept that includes physical, psychological, social, and environmental aspects. General QOL can be used as a health indicator in individuals with and without health conditions, in large scale epidemiological studies, health surveys, or as an outcome criterion in clinical intervention studies [2]. For the different fields of application in the context of health research, it is important to have an economic screening instrument for the assessment of general QOL. According to this aim, the EUROHIS project developed a short instrument for the assessment of the general QOL based on the two most common measurements of QOL, the WHOQOL-100 with 100 items [3] and the WHOQOL-BREF with 26 items [4].

In 2006, a short version of eight items was published for the use of population surveys, with two analyses using three different datasets of the WHOQOL-100 and the WHOQOL-BREF. Subsequently, the newly developed EUROHIS-QOL with eight items was validated in a sample of ten countries (France, Germany, the United Kingdom, Lithuania, Latvia, Croatia, Romania, Slovakia, the Czech Republic, Israel) [2]. After the development of the EUROHIS-QOL it was stated that general QOL can be assessed with the 8-item version for an overview of the four domains of physical, psychological, social, and environmental QOL in health surveys as it showed good internal consistency and acceptable convergent and discriminant validity in various studies [2, 5–7]. However, the WHOQOL-BREF should be used when QOL is a key indicator in the study [8]. Internationally, the EUROHIS-QOL has been widely used studying the association between physical as well as mental health diseases and QOL. For example, it was recently applied in a population-based study in Finland examining the negative impact of obesity on QOL [9]. The EUROHIS-QOL has also been successfully implemented in a study of deaf individuals with intellectual disabilities [10] and in an intervention study for persons with intellectual disabilities [11]. Considering mental health aspects, it has recently been used in the context of the COVID-19 pandemic, for example, to study the QOL and mental health of people with physical diseases [12] and in elderly people suffering from loneliness [13]. The German version of the EUROHIS-QOL was last validated and standardized in the general population in 2004, including indicators of mental health such as somatoform disorders and chronic stress [5]. Since then it has been used for various studies in the general population, e.g., to validate other instruments [14]. It has also been shown that individuals with symptoms of chronic fatigue and somatization had lower QOL compared to the general population [15]. In special populations, for example, the EUROHIS-QOL has been used to assess the mental health status of refugees [16]. It is also used in a randomized controlled trial for the treatment of bulimia nervosa [17]. Concerning physical health, the EUROHIS-QOL has been used as an example to investigate QOL and mental health in patients with dementia [18] or after a stroke [19].

The aim of the present study was to update the psychometric properties of the German version of the EUROHIS-QOL and to validate it with respect to depressiveness and anxiety, the most frequent symptoms of mental disorders in the general population. We were using a representative sample of the German population and calculated new norm values.

## Methods

In a survey of a representative sample of the German population (N = 2,015) data were collected with the help of the demography research institute USUMA in autumn 2021. Following a random route approach, one person from households in 258 German regional areas was randomly selected. Face to face interviews were carried out following hygiene regulations as for example wearing facial masks and keeping physical distance. By comparisons with the Federal Statistical Office, the sample proved representative for the German general population regarding age, gender, and education.

The study was conducted in accordance with the Declaration of Helsinki and the survey was approved by the Ethics Committee of the Medical Faculty of the University of Leipzig (298/21-ek). Anonymity in responses was guaranteed and informed consent was obtained from all respondents. To be eligible for survey inclusion, participants had to be at least 16 years of age and have sufficient German language skills. The sample design was not based on an exact criterion, as our dataset should allow the analysis of different research questions with a wide range of effect sizes. Therefore, we set 2,500 participants as the sample size to obtain stable effect estimates. We collected a representative sample of the German population and presented the sociodemographic characteristics of the sample. We reported all data exclusions and all measures we used from the different survey instruments.

### Measures

For this study two questionnaires and sociodemographic data of the survey were used. Socio-demographic information (age, gender, migration background, level of education, profession, employment, household income, religious affiliation, family status, partnership, living environment, and property) were assessed during the face-to-face interview. Additional information was obtained via a questionnaire. Age was assessed by year and month of birth. To determine gender, participants were asked to choose between male, female, or diverse. Migration background was defined following the definition of the German Federal Statistical Office [20]. The level of education was assessed according to the German school system and equalized income was calculated according to the OECD guideline by dividing the household income through the square root of people living in the household [21].

#### EUROHIS-QOL

The EUROHIS-QOL is an 8 item self-report questionnaire derived from the WHOQOL-100 and the WHOQOL-BREF. Conceptually, it captures a physical, a psychological, a social, and an environmental domain with 8 items (overall QoL, general health, energy, daily living activity, self-esteem, social relationships, finances, and home). It does not contain opposing item formulations and assesses general QOL. Each item is scored on a 5-point Likert scale. A higher total score on all items indicates a higher quality of life. The initial EUROHIS pilot study 2000 and the 2001 EUROHIS field study reported good psychometric properties [8]. Further, a cross-cultural study showed satisfactory Cronbach alpha value of 0.83 for internal consistency [2]. In a previous study with a representative sample of the German population in 2004, the German version of the EUROHIS-QOL reported a Cronbach alpha value of 0.85 [5].

#### PHQ-4

For validation purposes, the self-report questionnaire PHQ-4 [22, 23] was used, which measures depressiveness and general anxiety. Items are to be answered on a 4-point Likert scale (“not at all” to “almost every day”) for the past 2 weeks. Two items (PHQ-2) measure depressiveness [24], with scores ranging from 0 to 6 and a score of 3 or more indicating clinically relevant depressiveness with a sensitivity of 79% and a specificity of 86% [25]. The PHQ-2 has a high reliability of *α* = 0.83 [26]. In this sample α was 0.82. Anxiety symptoms were measured with two items (GAD-2) [27]. The GAD-2 score ranges from 0 to 6, and scores of 3 and above indicate an anxiety disorder (e.g., generalized anxiety disorder, social phobia, or panic disorder), with a sensitivity of 65% and a specificity of 88% [28]. The reliability of the GAD-2 is acceptable with α = 0.75 [26]. In this sample α was 0.76.

### Statistical Analyses

The psychometric testing of the German version of the EUROHIS-QOL involved several statistical procedures. Central descriptive and psychometric parameters for the 8 items and the sum score were employed using classical psychometric theory. To test internal consistency, Cronbach’s alpha and McDonald’s Omega were used. Non-response rates were calculated and expected to be smaller than 5%. Taking the previous results form a German norm sample as orientation [5], floor effects were considered if more than 3% of the participants chose the lowest possible score. Ceiling effects were considered if more than 30% of the participants chose the lowest or the highest possible score. An exploratory and a confirmatory factor analysis were conducted. A multiple linear regression analysis was performed to examine the influence of sociodemographic and socioeconomic variables. Discriminant validity was examined by Pearson correlations with the PHQ-4 (PHQ-2 and GAD-2) and Wilcoxon-tests were used to compare participants with clinically relevant depression or anxiety symptoms to the other participants. Norm values were calculated by using percentiles. Significance for statistical tests was set at *p* < 0.05 (two-sided). All analyses were conducted using R version 4.0.3 (packages: tidyvers [29], psych [30], car [31]).

## Results

### Norm Sample

The sample was representative of the German population and comprised 2,515 persons. The mean age was 50.09 years (SD = 18.05) and 51.6% of the sample reported female gender. A migration background was stated by 14.4% of the participants, 71% stated a religious affiliation, 53% lived with a partner, and 6.9% were unemployed. Further characteristics of the sample are shown in Table 1.

**TABLE 1:** Sociodemographic characteristics of the sample, N = 2,515 (Mainz, Germany. 2022).

		M	SD
	Age	50.09	18.05
	Equivalized household income	2,015.05	1012.12
		N	%
Gender	Female	1,297	51.6
	Male	1,217	48.4
	Divers	1	0
Age	≤ 40 years	825	32.8
	41–60 years	906	36.0
	≥ 61 years	784	31.2
Migration	No migration background	2,152	85.6
	Second generation migrants	258	10.3
	First generation migrants	104	4.1
Equivalized household income	<1000 EURO	230	9.3
	>1000–2000 EURO	1,177	47.7
	>2000–3000 EURO	679	27.5
	>3000 EURO	384	15.5
Religious affiliation	Yes	1,777	71.0
Family status	Single	797	31.7
	Married/living together	1,001	39.9
	Married/not living together	44	1.8
	Divorced	404	16.1
	Widowed	265	10.6
Partnership	Living with a partner	1,317	53.0
	Not living with a partner	1,170	47.0
Living environment	City^a^	1,501	59.68
	Rural area	1,014	40.32
Property	Tenant	1,648	66.4
	Living in own property	833	33.6
Education	University/college degree	256	10.2
	University entrance qualification	415	16.6
	Secondary school diploma (10 years)	1067	51.5
	Secondary school diploma (9 years)	675	26.9
	Without secondary diploma	53	2.1
	Student	44	1.8
Profession	Freelance professions	53	2.1
	Self-employed	141	5.7
	Civil servant	116	4.7
	Self-employed farmer	7	0.3
	Employed	1,448	58.1
	Skilled worker	337	13.5
	Worker	238	9.5
	Never worked	153	6.1
Employment	Full-time employed (>35 h per week)	1,127	45.0
	Part-time employed (15–34 h per week)	294	11.7
	Hourly employed	59	2.4
	Not Employed	62	2.5
	Unemployed	111	4.4
	Vocational training	57	2.3
	Education	115	4.6
	Pensioner/retired/early retirement	668	26.6
	Voluntary service/parental leave	14	0.6

Notes: N, sample size (valid cases); M, mean; SD, standard deviation.

a The division into city and rural area followed the political municipality sizing (GKPol), participants were assigned to city if they lived in a municipality with more than 20,000 inhabitants.

### Descriptive Analyses

Table 2 shows the descriptive analyses for each item and the sum score of the EUROHIS-QOL. The missing data rate was very low with 0.1% for all but one item. Item 6 asking about personal relationships had a missing response rate of 0.4%. For the sum score the missing response rate also was lower than 1%. No floor effects were found. However, all but the first, fifth and seventh item showed small ceiling effects with 31%–33% of participants rating the highest possible scores. The item frequency distributions across the five response categories showed that there was skewing of the data to the positive end of the response scales. A negative skewness was also found for the sum score. The mean values of the items vary slightly around 4 (good QOL). The mean value for item 7 (financial resources) deviates slightly downward and for item 8 (living place) deviates slightly upward. The mean value of the sum score was 31.66 and the median was 32. Person values do not fully exhaust the range of theoretically possible index values of 8–40, the lowest score was 12.

**TABLE 2:** Descriptive item characteristics for the German version of the quality of life questionnaire, N = 2 515 (Mainz, Germany. 2022).

Item	N	MV	M	SD	1	2	3	4	5
		N (%)			N (%)	N (%)	N (%)	N (%)	N (%)
1–How would you rate your quality of life	2 511	4 (0.1)	3.89	0.78	9 (0.4)	113 (4.5)	518 (20.6)	1,380 (55.0)	491 (19.6)
2–How satisfied are you with your health	2 512	3 (0.1)	3.90	0.95	44 (2.0)	213 (9.9)	357 (14.2)	1,229 (57.0)	669 (31.0)
3–Do you have enough energy for everyday life	2 512	3 (0.1)	4.01	0.85	4 (0.2)	123 (4.9)	503 (20.0)	1,087 (43.3)	795 (31.6)
4–How satisfied are you with your ability to perform your daily activities	2 511	4 (0.1)	4.01	0.84	9 (0.4)	160 (7.3)	332 (13.2)	1,306 (59.9)	704 (32.3)
5–How satisfied are you with yourself	2 512	3 (0.1)	3.97	0.81	12 (0.5)	157 (7.2)	324 (12.9)	1,418 (64.8)	601 (27.5)
6–How satisfied are you with your personal relationships	2 504	11 (0.4)	4.02	0.89	28 (1.3)	148 (6.9)	350 (14.0)	1,203 (48.0)	775 (31.0)
7–Have you enough money to meet your needs	2 512	3 (0.1)	3.77	0.91	28 (1.1)	177 (7.0)	692 (27.5)	1,064 (42.4)	551 (21.9)
8–How satisfied are you with the conditions of your living place	2 511	4 (0.1)	4.09	0.84	12 (0.5)	148 (5.9)	270 (10.8)	1,255 (50.0)	826 (32.9)
Sum score	N	MV (%)	M	SD	Md	Min.	Max.	Skew	Kurtosis
					N (%)	N (%)	N (%)		
Item 1–8	2 493	22 (0.87)	31.66	5.25	32	12	40	−0.69	0.36
					332 (13.32)	1 (0.04)	135 (5.42)		

Notes: N, sample size (valid cases); MV, proportion of missing values; M, mean; SD, standard deviation.

### Psychometric Properties

The psychometric properties of the EUROHIS-QOL items can be rated as good to very good. The discriminatory power (part whole corrected item-total correlation) is greater than 0.40 for all items (rIT = 0.48–0.72) and all items of the EUROHIS-QOL correlate moderately to highly with each other. Table 3 displays the psychometric item characteristics and item intercorrelations. The EUROHIS-QOL showed good internal consistency (*α* = 0.89; *ω* = 0.93)*.* When an item was deleted, Cronbach’s alpha coefficient ranged from 0.86 to 0.89 and McDonald’s omega coefficients from 0.92 to 0.94, indicating that each item is relevant for the instrument. The Kolmogorov-Smirnov test showed that the values of the sum score are not normally distributed (KST = 0.97, *p* = < 0.000). Guttman’s coefficient for test split-half reliability was high (λ6 = 0.91).

**TABLE 3:** Psychometric item characteristics and item intercorrelations for the German versions of the quality of life questionnaire, N = 2 515 (Mainz, Germany. 2022).

Item	rII											
	α-I	ω-I	rIT	*r* ^2^	1	2	3	4	5	6	7	8
	α = 0.89	ω = 0.93										
1	0.86	0.92	0.68	0.46	1							
2	0.87	0.92	0.66	0.43	0.56	1						
3	0.86	0.92	0.69	0.47	0.57	0.75	1					
4	0.87	0.92	0.71	0.50	0.57	0.72	0.78	1				
5	0.87	0.92	0.72	0.51	0.62	0.63	0.66	0.69	1			
6	0.88	0.94	0.59	0.26	0.54	0.45	0.46	0.49	0.62	1		
7	0.88	0.94	0.51	0.26	0.61	0.34	0.42	0.42	0.46	0.44	1	
8	0.89	0.94	0.48	0.23	0.55	0.31	0.36	0.38	0.42	0.45	0.60	1

Notes: α-I = Cronbach’s alpha of index when item is excluded; ω-I = McDonald’s omega of index when item is excluded; rIT, item-total correlation (part-whole corrected); *r* ^*2*^ = squared multiple correlation; rII, inter-item correlation; *α* = Cronbach’s alpha of the index; *ω* = McDonald’s omega of the index.

### Factor Validity

In the first step of the exploratory factor analysis, a two-factor solution was identified according to Velicer’s minimum average partial (MAP) test, eigenvalues, and parallel analysis. The factor loading matrix after maximum likelihood factor analysis with varimax rotation is shown in Supplementary Table S1. The cumulative total variance resolution was 64.3%. In the two-factor solution, one factor is constituted by items 2 to 5, which elucidates 36.8% of the total variance. The second factor comprises items 1 and 6 to 8. In terms of content, the first factor includes part of the physical and social domains, as well as the psychological dimension of the questionnaire. The second factor includes the environmental dimension, the question about the general quality of life, and about satisfaction with relationships. Thus, the two factors cannot be separated according to the dimensions that exist due to content considerations. Alternatively, it would be possible to assign items 1 and 6 with acceptable factor loadings <0.4 to factor 1, so that the factors can be described in terms of content along the specific dimensions of quality of life: Factor I takes into account the physical, psychological and social dimensions, Factor II the environmental dimension of quality of life. Due to the violation of the hypothesized one-factor-model a confirmatory factor analysis was performed by specifying one factor to be extracted. The total variance resolution of the factor was 53.5%. The factor loadings varied between 0.52 and 0.85 and are shown in the Supplementary File. At a critical level of 0.40 all factor loadings are considered good. The overall variance elucidation is slightly more than 10% below the two-factor solution.

### Multiple Linear Regression

A multiple linear regression was conducted to examine the influence of sociodemographic variables on the QOL sum score (Table 4). Overall, the included variables explained 30% of the variance of QOL. The statistically most important predictor for lower QOL was unemployment. Additionally younger age, not living with a partner, and having a migration background were statistically significant for the prediction of lower QOL. Higher QOL was associated with higher income, living in a city, living in own property, and having high education. Gender and religious affiliation were not statistically relevant predictors of QOL.

**TABLE 4:** Multiple linear regression analyses of quality of life on sociodemographic and socioeconomic characteristics, N = 2 365 (Mainz, Germany. 2022).

	Beta	SE	t value	*p*
Age	−0.30	0.02	−16.68	<0.000***
Gender (female)	0.00	0.03	0.04	0.970
Migration background (yes)	−0.20	0.05	−3.95	<0.000***
Equivalized household income	0.21	0.02	11.01	<0.000***
High education (yes)	0.09	0.04	2.13	0.034*
Living in a city (yes)	0.11	0.04	2.98	0.002**
Living in own property (yes)	0.34	0.04	8.59	<0.000***
Being a religiously affiliated (yes)	0.04	0.04	1.06	0.290
Unemployment (yes)	−1.20	0.09	−13.73	<0.000***
Not living with partner (yes)	−0.24	0.04	−6.64	<0.000***
Adjusted *R* ^2^	0.30			

Note. Statistically significant predictors are printed in bold; significance levels: *p < 0.05, **p < 0.01, ***p < 0.001; F-statistic: 85.99 on 12 and 2352 DF, *p*-value: < 0.000.

### Discriminant Validity

To examine discriminant validity intercorrelations between the EUROHIS-QOL sum score and its single items and PHQ-2 (depressiveness), GAD-2 (anxiety), and PHQ-4 were calculated (Table 5). The QOL sum score showed a high negative correlation with anxiety, depressiveness, and the overall PHQ-4. All items showed moderate, negative correlation with anxiety, except item 7 and 8 referring to the environmental QOL showing only low correlations. Correlations with depressiveness were negative and high for items 2 to 5 and moderate for items 1, and 6 to 8. The same applied for the correlations with PHQ-4. Discriminant validity between participants who reported clinically relevant scores in anxiety and depressiveness and participants who scored below the cut-offs was examined by Wilcoxon rank sum tests with continuity correction as the QOL data for participants below the cut-off were not normally distributed. Regarding depressiveness, the mean for QOL for persons with clinically relevant scores was 23.6 (N = 217) in comparison to 32.4 (N = 2 298) for the other participants. The Wilcoxon test showed that the two groups differed significantly in their QOL score with a small effect size (W = 438,627, *p* < 0.001, *r =* 0.16). For anxiety, the mean for QOL for persons with clinically relevant scores was 24.6 (N = 193) in comparison to 32.3 (N = 2 319) for the other participants. The Wilcoxon test showed that the two groups differed significantly in their QOL score with a small effect size (W = 375,532, *p* < 0.001, *r* = 0.16). Thus, the participants without clinically relevant scores for depressiveness and anxiety showed significantly higher QOL than the comparison groups. The strong negative correlations and the results of the group comparisons indicate a good discriminant validity of the EUROHIS-QOL.

**TABLE 5:** Correlations of quality of life with depressiveness and anxiety, (Mainz, Germany. 2022).

EUROHIS-QOL (Items and sum score)	GAD-2 (Anxiety)	PHQ-2 (Depressiveness)	PHQ-4
QOL sum score	−0.521	−0.625	−0.622
1–Overall QOL	−0.390	−0.460	−0.460
2–Satisfaction with health	−0.420	−0.510	−0.500
3–Satisfaction with energy for everyday life	−0.490	−0.570	−0.570
4–Satisfaction with daily activities	−0.470	−0.570	−0.570
5–Satisfaction with self	−0.480	−0.570	−0.570
6–Satisfaction with relationships	−0.400	−0.470	−0.470
7–Satisfaction with money	−0.290	−0.360	−0.350
8–Satisfaction with living place conditions	−0.270	−0.330	−0.330

### Norm Values

Norm values are reported for different age groups (<40 years, 41–60 years, > 60 years), for the total sample and separately for men and women in Table 6.

**TABLE 6:** Norm values (percentile ranks) separated by gender and age groups, N = 2 515 (Mainz, Germany. 2022).

Age	Total			Men			Women		
	<40	41–60	>60	<40	41–60	>60	<40	41–60	>60
	*n* = 820	*n* = 895	*n* = 778	*n* = 402	*n* = 445	*n* = 361	*n* = 418	*n* = 450	*n* = 416
Raw score (8–40)									
<15	0	0	0	0	0	0	0	1	0
16	0	1	1	0	0	1	0	1	1
17	1	1	1	1	1	2	0	1	1
18	1	2	2	1	2	3	1	2	2
19	1	3	3	1	3	3	1	3	3
20	2	4	4	1	4	4	2	4	4
21	2	5	6	2	5	6	3	5	6
22	3	7	8	2	7	8	4	7	8
23	4	8	11	3	9	11	5	8	11
24	5	10	14	4	11	15	6	10	13
25	7	12	17	6	13	18	8	12	16
26	9	14	21	7	15	21	10	14	20
27	11	17	25	9	18	25	12	16	25
28	13	20	30	11	21	31	15	19	30
29	16	24	36	14	26	37	18	23	36
30	21	28	44	19	30	43	22	27	44
31	27	35	51	26	36	50	28	33	52
32	37	46	62	35	48	61	39	44	62
33	47	57	72	44	58	73	49	56	72
34	55	65	80	52	66	80	58	65	79
35	63	72	86	60	73	85	66	72	86
36	70	79	90	66	80	89	73	78	91
37	77	84	93	73	85	93	81	83	94
38	84	88	95	80	89	95	87	87	95
39	89	92	97	87	93	97	92	92	97
40	100	100	100	100	100	100	100	100	100

## Discussion

Like previous national and international studies [2, 5–7], our findings indicate good psychometric properties of the German version of the EUROHIS-QOL, which was used in a representative sample of the German population in 2021. The missing values rates were low, the discriminatory power was good, and the intercorrelations of all items were moderate to high. The EUROHIS-QOL showed good internal consistency (*α* = 0.89; *ω* = 0.93), even when an item was deleted. Similar to the results from the German representative sample of 2004 the assumption of structural unidimensionality could not be fully confirmed. At least 2 of the 8 items of the index (“money” and “housing conditions”) formed a second factor as an exploratory factor analysis showed. Consequently, the two items capturing the environment-related dimension according to the four specific facets of quality of life of the original WHOQOL approach formed another factor and showed a significant amount of inherent variance compared with the items of the other three dimensions. However, with a loss of explained variance of about 10%, the results also point to the possibility of combining all eight items into one index (factor). Thus, compared to the results from the German representative sample of 2004, the psychometric properties remained stable [5]. In the context of the corona pandemic, it might have been expected that quality of life was negatively affected. However, in the second year of the pandemic, the QOL life in the German general population was higher on average than in 2004 [5]. The mean score of the total score increased from 30.99 to 31.66. Similarly, the mean scores of the individual items increased. It is important to note that the scores of all eight items and the sum score were not normally distributed, but showed a skew toward the positive end of the scale. This indicates a high level of satisfaction with QOL in the German general population and is consistent with previous results from studies testing the psychometric properties of the EUROHIS-QOL in Germany and other Western European countries [2]. As in 2004, satisfaction with financial resources had the lowest scores, while the other environmental element, living space, had the highest mean satisfaction score. Satisfaction with health, self, energy, and daily activities were in the good satisfaction range. In contrast, an Israeli study (N = 571) during the Corona pandemic found low QOL scores in the physical, psychological, and social domains of the WHOQOL-BREF, while the environmental domain was not affected. Quality of life was particularly low among women, younger adults, and the unemployed [32].

Sociodemographic predictors explained 30% of the variance in QOL in this study as well, indicating that the inclusion of these variables in QOL studies is essential to avoid overlooking substantial differences between population groups. Important predictors of lower QOL were unemployment, younger age, not living with a partner, and a migration background. A higher QOL was associated with a higher income, living in one’s own home, and a high level of education. Interestingly, in contrast to 2004, no gender differences were found. More recent national and international studies conducted during the Corona pandemic provided mixed results. In a German online study (N = 541), men reported higher overall quality of life. Individuals living alone reported lower scores, whereas those with higher education and age reported higher scores [33]. Using the WHOQOL-BREF in an online survey during home quarantine in China (N = 2 289), sex differences were only found for the environmental domain, with men reporting higher QOL [34]. In a population-based online survey in Italy (N = 2 251), women reported lower QOL in the physical, psychological, and environmental domains of the WHOQOL-BRef. [35]. No gender differences were found in a Saudi Arabian online survey sample (N = 1,029), that used the EUROHIS-QoL. In this study social support contributed to higher QOL during the pandemic, whereas fear of COVID-19 only indirectly influenced QOL via lower mental well-being [36]. Beyond sociodemographic predictors, other factors such as social support and loneliness [12] showed strong associations with QOL and should be considered in future studies.

With respect to mental health, our results showed good discriminant validity of the EUROHIS-QOL for anxiety and depression symptoms measured by the PHQ-4 by the strong negative correlations and the results of group comparisons. Participants without clinically relevant scores for depressiveness and anxiety showed significantly higher QOL. Inter-item correlations were higher for depressiveness than for anxiety. Previous studies examining depressed individuals with the EUROHIS-QOL showed similar results and underscored the negative association between clinical depression and quality of life measured with the EUROHIS-QOL [7, 18, 19].

Benefits of the study were the availability of data of a representative face-to-face survey which included information about participants’ personal and socio-demographic characteristics. However, the results need to be interpreted considering the study’s limitations. The cross-sectional design does not allow drawing conclusions regarding the direction of associations. Additionally, the focus of the sampling methods was to ensure a sample representative of the German general population regarding age, gender, and education. This, however, does not allow to provide information on vulnerable groups, e.g., minorities or clinical populations.

### Conclusion

In summary, the EUROHIS-QOL is an economical and reliable instrument for assessing QOL in the German general population. As unidimensional and short instrument with eight items, it is particularly useful for population-based surveys and other studies when longer instruments cannot be used.
