# Supplementary material for: Quality of Life: Updated Psychometric Properties and New Norm Values in a Representative German Sample Focusing Socioeconomics and Mental Health
Source: Int J Public Health. 2022 Oct 5;67:1605188. doi: 10.3389/ijph.2022.1605188 (PMC9579288; doi:10.3389/ijph.2022.1605188)
Supplement: Supplementary file 1 [file Table1.pdf]

Supplementary File

Table 1: Results of exploratory and confirmatory factor analyses for the quality of life questionnaire, N = 2,493 (Mainz, Germany. 2022).

Modell	EFA		CFA
Item	Factor I	Factor II	Factor I
1	0.47	0.66	0.74
2	0.80	0.23	0.80
3	0.84	0.28	0.85
4	0.82	0.30	0.85
5	0.66	0.45	0.81
6	0.42	0.52	0.63
7	0.23	0.74	0.57
8	0.18	0.72	0.52
Eigenvalue	2.940	2.206	4.279
Variance	0.368	0.276	0.535
Cumulative variance	0.368	0.643	
Chi square value	316.17		1802.91
df	13		20
p-value	< .000		0

Notes: EFA = Exploratory factor analysis; CFA = Confirmatory factor analysis
